# Impact of Tumor Volume and Other Factors on Renal Function After Partial Nephrectomy

**DOI:** 10.3390/jcm13216305

**Published:** 2024-10-22

**Authors:** Ignacio González-Ginel, Mario Hernández-Arroyo, Clara García-Rayo, Carmen Gómez-del-Cañizo, Alfredo Rodríguez-Antolín, Félix Guerrero-Ramos

**Affiliations:** Department of Urology, Hospital Universitario 12 de Octubre, 28045 Madrid, Spain; mario.hernandez@salud.madrid.org (M.H.-A.); clara.garciarayo@salud.madrid.org (C.G.-R.); cgdelcanizo@salud.madrid.org (C.G.-d.-C.); alfredo.rodriguez@salud.madrid.org (A.R.-A.); felixguerrero@gmail.com (F.G.-R.)

**Keywords:** partial nephrectomy, renal cell carcinoma, chronic kidney disease

## Abstract

**Background and Objectives**: One of the main objectives of partial nephrectomy is to preserve as much renal function as possible. However, the removal of functioning nephrons and ischemic damage secondary to the clamping of the renal pedicle can be associated with a certain degree of renal function loss. We aim to evaluate the relationship between tumor volume and other factors on renal function in the short term (1–3 months) and long term (6–12 months) in our series of partial nephrectomies. **Methods**: A retrospective study was conducted on 147 patients who underwent open or laparoscopic partial nephrectomy between 2018 and 2022. Tumor volume was estimated through reconstructions from the computed tomography images. Univariate and multivariate analyses of the data were performed. **Results**: The mean age was 58.2 years, with an average glomerular filtration rate (GFR) of 79 mL/min/m^2^. Of all partial nephrectomies, 76.2% were laparoscopic, 57.1% were T1a tumors, and the mean volume was 17 cc. The average ischemia time during surgery was 14.3 min, with a mean hemoglobin loss of 2.8 g/dL in the immediate postoperative period. No significant differences were found either in the short or long term regarding ischemia time (*p* = 0.57, *p* = 0.32) or tumor volume (*p* = 0.57, *p* = 0.33). However, in the multivariate analysis, it was observed that the variable with the greatest influence on short-term renal function was perioperative glomerular filtration, while in the long term, ischemia time, age, and tumor volume also had an influence. **Conclusions**: Tumor volume is not an independent factor for renal function deterioration in the short or long term. Short-term renal function is primarily determined by perioperative glomerular filtration. Perioperative glomerular filtration, ischemia time, age, and tumor volume can jointly predict long-term renal function.

## 1. Introduction

Renal cell carcinoma (RCC) represents approximately 3% of all malignancies globally and is the most common type of kidney cancer, with clear cell renal cell carcinoma (ccRCC) being the predominant subtype, accounting for about 70% of cases. The incidence of RCC has been steadily increasing worldwide, with an estimated 400,000 new cases annually, contributing to a significant global health burden [1,2].

The standard treatment for localized RCC has traditionally been radical nephrectomy (RN). However, the understanding of RCC’s natural history and the development of nephron-sparing techniques have shifted the treatment paradigm towards partial nephrectomy (PN), especially for smaller tumors (T1a and T1b) [3]. PN aims to achieve oncological control while preserving as much renal function as possible, thus reducing the risk of chronic kidney disease (CKD) and its associated morbidity and mortality [4,5]. The preservation of renal function is particularly important as CKD has been associated with an increased risk of cardiovascular events and all-cause mortality [5,6].

Despite the advantages of PN, the procedure is not without challenges. Several factors, including tumor size, location, ischemia time, and patient-related factors such as age and baseline renal function can influence the postoperative functional outcomes [6]. Tumor volume has been suggested as a potential predictor of renal function decline after PN, but its independent impact remains controversial [7]. Larger tumors may require a more extensive resection of the renal parenchyma, potentially leading to a greater loss of nephrons and a subsequent decline in renal function [8]. However, some studies have found that tumor volume alone may not be a significant predictor of renal function outcomes, suggesting that other factors may play a more critical role [9,10].

In terms of oncological safety, PN has demonstrated equivalent cancer-specific survival rates compared to RN in small renal masses, with some studies even showing a reduced risk of cardiovascular events and better overall survival in patients undergoing PN versus RN [10,11]. For larger tumors (T2), while the use of PN is more controversial, advances in surgical techniques and perioperative management have allowed for its safe application in select cases, with favorable outcomes [12]. This shift towards a nephron-sparing approach reflects an increasing recognition of the importance of renal function preservation as a key determinant of long-term patient outcomes.

Ischemia time during PN is another important factor that can affect postoperative renal function [11]. Prolonged ischemia can lead to ischemia-reperfusion injury, resulting in acute kidney injury (AKI) and potentially progressing to CKD [12]. Minimizing ischemia time, therefore, is crucial in reducing the risk of renal function deterioration, especially in patients with pre-existing CKD or reduced baseline renal function [13]. Techniques such as “off-clamp” PN or selective arterial clamping have been developed to reduce the warm ischemia time and have been shown to significantly improve postoperative renal function outcomes [14]. These techniques, while technically demanding, are increasingly favored in cases where even short periods of ischemia could lead to substantial postoperative renal function impairment.

Furthermore, PN remains a technically demanding procedure, particularly for tumors located near critical structures such as the renal hilum or collecting system. Tumors in these challenging locations require careful surgical planning to balance the need for oncological control with the preservation of renal function. Complex cases may necessitate the use of advanced imaging modalities, such as three-dimensional reconstructions, to provide a more detailed understanding of the tumor’s relationship with the surrounding structures. These reconstructions can assist in preoperative planning and enhance the surgeon’s ability to perform precise, nephron-sparing resections [15,16]. Intraoperative imaging techniques, such as ultrasound guidance, have also become valuable tools for delineating tumor margins and minimizing damage to healthy tissue during resection [17]. This is particularly important in cases where the tumor is endophytic, making it difficult to visualize the entire lesion.

Age is another critical factor that can influence renal outcomes after PN. Older patients are more likely to have a reduced baseline renal function and a higher prevalence of comorbidities such as hypertension and diabetes, which can postoperatively exacerbate the decline in renal function [14,15]. The aging kidney is also more susceptible to ischemic injury due to structural and functional changes, such as glomerulosclerosis and reduced renal blood flow [16]. As a result, elderly patients undergoing PN may be at a higher risk of experiencing both short- and long-term declines in renal function. Special consideration must be given to these patients during surgical planning to minimize ischemia time and preserve renal function as much as possible.

Baseline renal function, typically assessed by an estimated glomerular filtration rate (eGFR), is a well-established predictor of postoperative renal outcomes. Patients with a lower preoperative eGFR are at a higher risk of a significant decline in renal function after PN, as they have less functional renal reserve to compensate for the loss of nephron mass [17]. The preservation of renal function is particularly critical in these patients to prevent the progression to CKD and its associated complications [18]. Additionally, the postoperative recovery of renal function can vary widely depending on the extent of nephron loss, ischemia time, and the patient’s overall health. Monitoring and managing these factors are key to ensuring optimal long-term outcomes after PN.

Given the complexity of factors influencing renal outcomes after PN, understanding the interaction between tumor volume, ischemia time, age, and baseline renal function is essential for optimizing surgical outcomes and preserving renal function. This study aims to evaluate the impact of these factors on renal function in both the short- and long term following PN, using data from a cohort of patients treated at an academic tertiary care center to help inform clinical decision making and guide the selection of patients who would benefit most from nephron-sparing surgery.

## 2. Materials and Methods

We present a retrospective, observational analysis conducted on patients who underwent PN at our Tertiary Referral Academic Center between 2018 and 2022. Given the observational nature of the study, an exemption status was requested and confirmed by the ethics committee of our center, ensuring adherence to ethical research standards.

### 2.1. Study Population

The inclusion criteria were as follows:

- Patients aged 18 years or older.
- Patients with confirmed RCC and who underwent PN as the primary surgical intervention.
- Availability of preoperative and postoperative renal function data, specifically serum creatinine levels and eGFR values.
- Tumor volume accurately estimated through preoperative imaging techniques, including computed tomography (CT) with volumetric reconstructions.

Exclusion criteria were as follows:

- Patients who underwent radical nephrectomy instead of PN.
- Incomplete or inadequate medical records or patients without sufficient follow-up data, particularly postoperative renal function tests.
- Patients with metastatic disease or those who had previously undergone nephrectomy on the contralateral kidney, as this could skew renal function results.

### 2.2. Data Collection

The data were retrieved from the electronic medical records of the hospital’s internal system. The dataset included demographic variables such as age, gender, and body mass index (BMI). Additionally, medical histories were reviewed for comorbidities like hypertension and diabetes, which are known to influence renal function outcomes. Renal function was assessed using the eGFR based on serum creatinine levels, and calculated using the CKD-EPI equation.

Surgical details were documented, including ischemia time, which was defined as the total duration of the renal pedicle clamping. Tumor volume was calculated using preoperative CT imaging, which provided three-dimensional volumetric reconstructions of the renal mass. Tumor characteristics, such as histology, location, and staging, were also collected.

Postoperative renal function was assessed in both the short-term (1–3 months) and long-term (6–12 months) intervals. If multiple eGFR determinations were available during these periods, the mean value was calculated for a more accurate representation. Patients with incomplete follow-up data were excluded from the analysis to maintain consistency in the dataset. Renal function deterioration was defined as a significant decrease in eGFR or a substantial increase in serum creatinine levels from baseline, following the guidelines used in previous literature.

### 2.3. Statistical Analysis

Descriptive statistics were used to summarize the demographic and clinical characteristics of the study population. Continuous variables such as age, tumor volume, and ischemia time were expressed as means with standard deviations, while categorical variables like gender, tumor stage, and surgical technique were presented as frequencies and percentages.

Univariate analysis was performed to identify the variables potentially associated with changes in postoperative renal function. This analysis used independent t-tests for continuous variables and chi-square tests for categorical variables. Variables that showed significant associations with renal function outcomes were then included in a multivariate linear regression model to assess their independent impact, particularly focusing on the influence of tumor volume, ischemia time, and preoperative renal function.

A *p*-value of <0.05 was considered statistically significant for all analyses. All data analyses were performed using SPSS software version 26.0 for Windows (Chicago, IL, USA). Missing data were handled by excluding cases where critical variables such as ischemia time or follow-up renal function were not available.

## 3. Results

### 3.1. Patient and Tumor Characteristics

Our cohort included data from 147 subjects, comprising 98 males (66.7%) and 49 females (33.3%), with a mean age of 58.2 years. The average BMI was 28.2 kg/m^2^ (Table 1). Hypertension was the most common comorbidity, affecting 46.3% of patients, while 16.3% had a history of diabetes mellitus. Additionally, ischemic heart disease and CKD were each observed in 3.4% of the cohort (Table 2).

Tumor characteristics varied across the study group, with a mean tumor volume of 16.99 cm^3^, ranging from 0.44 to 144 cm^3^. Most tumors (59.9%) were classified as pT1a, indicating early stage, localized disease. Additionally, 2.7% of the tumors exhibited an endophytic growth pattern, which can present challenges for surgical resection. Regarding tumor histology, the most common type was clear cell RCC (49.7%), followed by papillary RCC (19.7%), and oncocytomas (14.3%). Chromophobe RCC represented 6.8%, while metastases were rare, occurring in only 0.7% of cases (Table 2).

The location of the tumors was evenly distributed between the right (44.9%) and left kidney (55.1%). Most tumors were in the upper (36.7%) and lower poles (34%) of the kidney, while the middle anterior and posterior locations were less common (19.7% and 7.5%, respectively).

### 3.2. Renal Function Outcomes

Postoperative renal function was assessed at both short-term and long-term intervals. The mean eGFR at discharge was 73 mL/min/1.73 m^2^, indicating a modest decline from the preoperative value of 79 mL/min/1.73 m^2^. The long-term follow-up (6–12 months) showed a slight improvement, with a mean eGFR of 74 mL/min/1.73 m^2^ (Table 1).

### 3.3. Univariate Analysis

Univariate analysis identified several factors associated with renal function outcomes. Older age (*p* < 0.001) and higher perioperative serum creatinine levels (*p* < 0.001) were strong predictors of worse postoperative renal function. However, ischemia duration and tumor volume were not significantly associated with renal function outcomes in either the short-term (*p* = 0.754 and *p* = 0.566, respectively) or long-term follow-up (*p* = 0.259 and *p* = 0.324, respectively). Tumor location was not a significant predictor of renal function outcomes, with *p* = 0.220 for short-term outcomes and *p* = 0.903 for long-term outcomes.

Hypertension (*p* = 0.004) and pre-existing CKD (*p* = 0.01) were significant predictors of poorer renal outcomes. Other comorbidities, including diabetes mellitus, ischemic heart disease, and high BMI, were not significant (*p* > 0.05).

The type of surgical approach (open vs. laparoscopic) was associated with differences in long-term renal function, with open surgery showing a trend towards greater renal function decline (*p* = 0.057) (Table 3).

### 3.4. Multivariate Analysis

The multivariate analysis confirmed that perioperative eGFR was found to be the strongest predictor of postoperative renal function. Patients with a higher preoperative eGFR had better preservation of renal function post-surgery. The regression model demonstrated that a decrease in preoperative and postoperative eGFR had a worsening effect on both short-term (*p* = 0.004) and long-term eGFR (*p* < 0.001).

Ischemia time was another significant predictor, particularly for long-term renal function outcomes. The analysis showed that longer ischemia times were associated with greater declines in eGFR at the 6- to 12-month follow-up (*p* = 0.036). Ischemia time did not reach statistical significance for short-term renal function (*p* = 0.173).

Age also played a role in predicting renal outcomes, particularly in the long term. Older patients experienced greater declines in eGFR, with age showing a correlation with long-term outcomes. Although age did not have a statistically significant effect on either short- or long-term eGFR (*p* = 0.964, *p* = 0.119), its influence became more pronounced over time as patients aged in the multivariate model.

**Figure 1:**
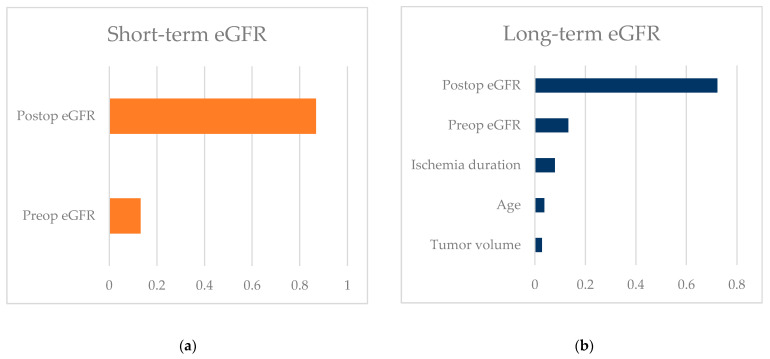
Impact of the predictive factors for renal function in the multivariate model: (**a**) in the short-term; and (**b**) in the long-term.

Tumor volume was not found to be an independent predictor of renal function decline in either short-term (*p* = 0.465) or long-term (*p* = 0.337) analysis, although it had a minor impact in predicting long-term eGFR (Table 4, Figure 1).

## 4. Discussion

The findings of our study indicate that tumor volume, although a critical factor in the surgical management of RCC, does not independently predict postoperative renal function decline following PN. This conclusion aligns with several previous studies that have demonstrated that other factors, particularly ischemia time and baseline renal function, play more significant roles in determining renal outcomes [14,15,19]. The results suggest that, while tumor volume may necessitate a more extensive resection, it is the cumulative effect of multiple factors, rather than tumor volume alone, that influences postoperative renal function.

Although previous studies have highlighted tumor size as a key predictor of renal function decline after surgery, especially for tumors larger than 17 cm^3^ or with a diameter greater than 4 cm, our findings challenge this view [20,21,22]. Through a multifactorial analysis, we demonstrate that tumor volume is not an independent predictor when other factors, such as ischemia time, age, and preoperative renal function, are considered. This distinction sets our study apart, offering a more nuanced perspective on renal function preservation following partial nephrectomy.

The lack of a significant association between tumor volume and renal function decline in this study may be explained by the relatively small size of the tumors in the cohort, with most being classified as T1a lesions. These smaller tumors are more likely to be managed effectively with nephron-sparing surgery techniques, minimizing the impact on renal function [23]. Moreover, the preservation of renal parenchyma, even in the presence of larger tumors, is increasingly achievable with advancements in surgical techniques, such as off-clamp or zero-ischemia PN, which aim to reduce the ischemic insult to the kidney [24,25].

Ischemia time, on the other hand, was found to be a significant predictor of renal function decline, particularly in the long term. This finding is consistent with the literature, which has shown that prolonged ischemia during PN is associated with an increased risk of AKI and subsequent CKD [11,13]. The duration of ischemia is a crucial determinant of the extent of renal injury [13,26]. In this study, efforts to minimize the ischemia time were associated with a better preservation of renal function, underscoring the importance of surgical techniques that reduce the need for the prolonged clamping of the renal artery [24].

Age was also identified as a significant factor influencing long-term renal outcomes. Older patients are more likely to experience a decline in renal function postoperatively, reflecting the reduced renal reserve and increased susceptibility to ischemic injury associated with aging [15,25]. This finding emphasizes the importance of considering patient age when planning PN, as older individuals may require more conservative surgical approaches or closer postoperative monitoring to prevent renal function deterioration [8,27]. The aging kidney’s reduced ability to recover from ischemic insult highlights the need for tailored surgical strategies that minimize ischemic exposure in this population [8].

Baseline renal function, as measured by eGFR, was the strongest predictor of both short-term and long-term renal outcomes in this study. Patients with lower preoperative eGFR experienced more significant declines in renal function after PN, consistent with previous studies [17,18,28]. We also included patients with pre-existing CKD to assess the deterioration in renal function within this subgroup, finding that CKD itself was an independent predictor of a greater decline in renal function. This finding draws attention to the relevance of the preoperative assessment of renal function in predicting postoperative outcomes and guiding surgical decision-making. For patients with compromised renal function, strategies to further preserve renal function, such as avoiding unnecessary nephron loss and minimizing ischemia time, are critical to improving long-term outcomes [7,28].

The results of this study have important implications for clinical practice. They suggest that a comprehensive preoperative assessment including the evaluation of tumor characteristics, patient age, baseline renal function, and surgical factors (such as planned ischemia time) is essential in order to optimize outcomes after PN. While tumor volume is crucial for surgical planning, it should not be viewed in isolation. Instead, it should be considered alongside other factors that collectively influence renal function [15,17].

Furthermore, our findings support the use of personalized surgical approaches that tailor the extent of resection and ischemia time to the individual patient’s risk profile. For example, in patients with small tumors and good baseline renal function, efforts to minimize ischemia time and preserve as much renal parenchyma as possible may result in better long-term renal outcomes [25,27]. Conversely, in patients with larger tumors or compromised renal function, more aggressive measures to reduce ischemic injury, such as the use of off-clamp techniques, may be warranted to prevent significant renal function decline, although these measures may also carry a higher risk of bleeding [24,28].

Despite the strengths of this study, including its sample size and comprehensive analysis of multiple factors influencing renal outcomes, several limitations should be acknowledged. The retrospective nature of this study may introduce selection bias, as patients with more complete follow-up data were more likely to be included in the analysis [27]. Additionally, the study’s reliance on eGFR as the primary measure of renal function may not capture all aspects of renal health, such as tubular function or proteinuria, which could also be impacted by PN [29].

Another limitation of our study is the lack of a detailed evaluation of comorbidities, nephrotoxic drugs, and different surgical techniques. Future research should aim to validate these findings in larger, prospective studies that include more diverse patient populations, longer follow-up periods, and address these additional factors. Investigating these aspects further could provide a more comprehensive understanding of the variables influencing renal outcomes. Moreover, the integration of novel biomarkers and imaging techniques could provide a more comprehensive assessment of renal function and help refine predictive models [30,31].

We believe that future research could benefit from integrating such technologies to further enhance predictive capabilities for renal outcomes after partial nephrectomy. Recent studies have demonstrated that machine learning algorithms can analyze large datasets to identify patterns and predict which patients are at higher risk for postoperative renal function decline. Incorporating these models in future work, alongside traditional clinical assessments, could improve decision-making processes and better identify patients who would benefit the most from nephron-sparing approaches [14，^17^,^18^].

In conclusion, tumor volume alone does not independently predict renal function deterioration in the short or long term. Instead, short-term renal function is primarily determined by perioperative glomerular filtration. For long-term outcomes, the combined effects of ischemia time, patient age, and baseline renal function play a crucial role. These findings highlight the importance of a personalized approach to surgical planning. By tailoring surgical strategies to the individual patient’s risk profile and leveraging emerging technologies, it may be possible to improve renal outcomes and reduce the long-term burden of CKD in patients undergoing PN.

## Figures and Tables

**Table 1:** Patient characteristics and tumor characteristics, quantitative variables.

Variable	Mean	Median	Standard Deviation	Minimum	Maximum
Age, years	58.24	59	13.84	27	85
BMI ^a^, kg/m^2^	28.27	28	5.5	18	46
Ischemia duration, min	14.33	14	7.61	0	40
Tumor volume, cm^3^	16.99	10.5	20.94	0.44	144
Hemoglobin loss, g/dL	2.27	2.1	1.14	0.2	4.2
Preoperative eGFR ^b^, mL/min/1.73 m^2^	79.06	85	14.85	24	90
Postoperative eGFR^2^, mL/min/1.73 m^2^	73.45	83.5	20.9	12	90
Short-term eGFR^2^ (mL/min/1.73 m^2^)	73.12	81	19.91	12	90
Long-term eGFR^2^ (mL/min/1.73 m^2^)	74.12	78.75	17.31	14	90

^a^ BMI: body mass index. ^b^ Estimated glomerular filtration rate. Short-term renal function was defined as the average of eGFR measurements between 1 and 3 months postoperatively, while long-term renal function was assessed using values obtained between 6 and 12 months after surgery.

**Table 2:** Patient characteristics and tumor characteristics, qualitative variables.

Variable		Percentage (Number)
Sex		
	Male	66.7% (98)
	Female	33.3% (49)
Hypertension		46.3% (68)
Diabetes mellitus		16.3% (24)
Ischemic heart disease		3.4% (5)
Chronic kidney disease		3.4% (5)
Surgical approach		
	Open	23.8% (35)
	Laparoscopic	76.2% (112)
Arterial clamping		
	Total	85% (125)
	Selective	4.8% (7)
	None	6.1% (9)
Side		
	Right	44.9% (66)
	Left	55.1% (81)
Location		
	Upper pole	36.7% (54)
	Lower pole	34% (50)
	Middle anterior	19.7% (29)
	Middle posterior	7.5% (11)
Endophytic		2.7% (4)
Histology		
	Clear cell	49.7% (73)
	Papillary	19.7% (29)
	Chromophobe	6.8% (10)
	Oncocytoma	14.3%
	Metastasis	0.7% (1)
	Other	8.8% (13)
pT		
	1a	59.9% (88)
	1b	6.1% (9)
	2c	0.7% (1)
	3a	1.4% (2)

**Table 3:** Univariate analysis of renal function after partial nephrectomy. In bold, statistically significant variables.

Variable	Short-Term eFGFR	Long-Term eFGFR
	*p*-Value	
Age	**0.001**	**0.001**
BMI ^a^	0.330	0.737
Ischemia duration	0.754	0.259
Tumor volume	0.566	0.324
Hemoglobin loss	0.983	0.219
Preoperative eGFR ^b^	**0.001**	**0.001**
Postoperative eGFR ^b^	**0.001**	**0.001**
Sex	0.615	0.941
Hypertension	**0.004**	**0.001**
Diabetes mellitus	0.521	0.122
Ischemic heart disease	0.776	0.073
Chronic kidney disease	**0.010**	**0.008**
Surgical approach	0.072	0.054
Arterial clamping	0.744	0.861
Side	0.107	0.965
Location	0.220	0.903
Endo/exophytic	0.797	0.797
Histology	0.369	0.066

^a^ BMI: body mass index. ^b^ Estimated glomerular filtration rate. Short-term renal function was defined as the average of eGFR measurements between 1 and 3 months postoperatively, while long-term renal function was assessed using values obtained between 6 and 12 months after surgery.

**Table 4:** Multivariate linear regression analysis of renal function after partial nephrectomy.

Variable	β ± Standard Error (*p*-Value)	
	Short-Term eGFR	Long-Term eGFR
Age	−0.004 ± 0.098 (0.964)	−0.114 ± 0.072 (0.119)
BMI ^a^	−0.075 ± 0.208 (0.719)	−0.023 ± 0.147 (0.875)
Ischemia duration	0.233 ± 0.169 (0.173)	0.265 ± 0.125 (0.036)
Tumor volume	0.058 ± 0.078 (0.465)	−0.056 ± 0.062 (0.337)
Hemoglobin loss	−0.696 ± 1.016 (0.496)	−0.458 ± 0.774 (0.556)
Preoperative eGFR ^b^	0.348 ± 0.117 (0.004)	−0.329 ± 0.092 (0.001)
Postoperative eGFR ^b^	0.553 ± 0.081 (0.001)	−0.514 ± 0.064 (0.001)

^a^ BMI: body mass index. ^b^ Estimated glomerular filtration rate. Short-term renal function was defined as the average of eGFR measurements between 1 and 3 months postoperatively, while long-term renal function was assessed using values obtained between 6 and 12 months after surgery.

## Data Availability

The data that support the findings of this study are available from the corresponding author upon reasonable request.
