# Correction: González-Ginel et al. Impact of Tumor Volume and Other Factors on Renal Function After Partial Nephrectomy. *J. Clin. Med*. 2024, *13*, 6305

**DOI:** 10.3390/jcm14103386

**Published:** 2025-05-13

**Authors:** Ignacio González-Ginel, Mario Hernández-Arroyo, Clara García-Rayo, Carmen Gómez-del-Cañizo, Alfredo Rodríguez-Antolín, Félix Guerrero-Ramos

**Affiliations:** Department of Urology, Hospital Universitario 12 de Octubre, 28045 Madrid, Spainfelixguerrero@gmail.com (F.G.-R.)

In the original publication [1], References [1,6,14,15,17–19,23–31] were incorrect. Errors occurred for some references due to mistakes made when formatting the citations according to the journal’s requirements. These oversights were unintentional and were discovered during a thorough review of the manuscript post-publication. All affected references have now been carefully cross-checked against the original sources and corrected. The corrections made do not alter the substance or conclusions of the article. Attached to this form is a correction document that includes the corrected versions of all the references, providing a comprehensive and accurate list of citations for the article. 

## References in the Original Publication

1. Bukavina, L.; Bensalah, K.; Bray, F.; Carlo, M.; Challacombe, B.; Karam, J.A.; Kassouf, W.; Mitchell, T.; Montironi, R.; O’Brien, T.; et al. Epidemiology of Renal Cell Carcinoma: 2022 Update. *Eur. Urol.* **2022**, *82*, 529–542. https://doi.org/10.1016/j.eururo.2022.08.019.
6. McKiernan, J.; Yossepowitch, O.; Kattan, M.W.; Simmons, R.; Motzer, R.J.; Reuter, V.E.; Russo, P. Partial Nephrectomy for Renal Cortical Tumors: Pathologic Findings and Impact on Outcome. *Urology* **2002**, *60*, 1003–1009. https://doi.org/10.1016/S0090-4295(02)01967-2.
14. Bhindi, B.; Lohse, C.M.; Schulte, P.J.; Mason, R.J.; Cheville, J.C.; Boorjian, S.A.; Leibovich, B.C.; Thompson, R.H. Predicting Renal Function Outcomes After Partial and Radical Nephrectomy. *Eur. Urol.* **2019**, *75*, 766–772. https://doi.org/10.1016/j.eururo.2018.11.021.
15. Nakamura, M.; Kameyama, S.; Ambe, Y.; Teshima, T.; Izumi, T.; Tsuru, I.; Inoue, Y.; Yoshimatsu, T.; Inatsu, H.; Amakawa, R.; et al. Predictive Factors for Postoperative Renal Function After Off-Clamp, Non-Renorrhaphy Partial Nephrectomy. *Transl. Androl. Urol.* **2022**, *11*, 1226–1233. https://doi.org/10.21037/tau-22-321.
17. Uleri, A.; Baboudjian, M.; Gallioli, A.; Territo, A.; Gaya, J.M.; Sanz, I.; Robalino, J.; Casadevall, M.; Diana, P.; Verri, P.; et al. A New Machine-Learning Model to Predict Long-Term Renal Function Impairment After Minimally Invasive Partial Nephrectomy: The Fundació Puigvert Predictive Model. *World. J. Urol.* **2023**, *41*, 2985–2990. https://doi.org/10.1007/s00345-023-04593-8.
18. Oh, S.W.; Byun, S.-S.; Kim, J.K.; Jeong, C.W.; Kwak, C.; Hwang, E.C.; Kang, S.H.; Chung, J.; Kim, Y.-J.; Ha, Y.-S.; et al. Machine Learning Models for Predicting the Onset of Chronic Kidney Disease After Surgery in Patients with Renal Cell Carcinoma. *BMC Med. Inform. Decis. Mak.* **2024**, *24*, 85. https://doi.org/10.1186/s12911-024-02473-8.
19. Yoon, H.-K.; Lee, H.-J.; Yoo, S.; Park, S.-K.; Kwon, Y.; Jun, K.; Jeong, C.W.; Kim, W.H. Acute Kidney Injury Adjusted for Parenchymal Mass Reduction and Long-Term Renal Function After Partial Nephrectomy. *J. Clin. Med.* **2019**, *8*, 1482. https://doi.org/10.3390/jcm8091482.
23. Tappero, S.; Bravi, C.A.; Khene, Z.E.; Campi, R.; Pecoraro, A.; Diana, P.; Re, C.; Giulioni, C.; Beksac, A.T.; Bertolo, R.; et al. Assessing Functional Outcomes of Partial Versus Radical Nephrectomy for T1b-T2 Renal Masses: Results from a Multi-Institutional Collaboration. *Ann. Surg. Oncol.* **2024**, *31*, 5465–5472. https://doi.org/10.1245/s10434-024-15305-w.
24. Schleef, M.; Roy, P.; Lemoine, S.; Paparel, P.; Colombel, M.; Badet, L.; Guebre-Egziabher, F. Renal and Major Clinical Outcomes and Their Determinants After Nephrectomy in Patients with Pre-existing Chronic Kidney Disease: A Retrospective Cohort Study. *PLoS ONE* **2024**, *19*, e0300367. https://doi.org/10.1371/journal.pone.0300367.
25. Saitta, C.; Garofano, G.; Lughezzani, G.; Meagher, M.F.; Yuen, K.L.; Fasulo, V.; Diana, P.; Uleri, A.; Piccolini, A.; Mancon, S.; et al. Preoperative Age and Its Impact on Long-Term Renal Functional Decline After Robotic-Assisted Partial Nephrectomy: Insights from a Tertiary Referral Center. *Medicina* **2024**, *60*, 463. https://doi.org/10.3390/medicina60030463.
26. Obrecht, F.; Padevit, C.; Froelicher, G.; Rauch, S.; Randazzo, M.; Shariat, S.F.; John, H.; Foerster, B. The Association of Ischemia Type and Duration with Acute Kidney Injury after Robot-Assisted Partial Nephrectomy. *Curr. Oncol.* **2023**, *30*, 9634–9646. https://doi.org/10.3390/curroncol30110698.
27. Ito, H.; Muraoka, K.; Uemura, K.; Jikuya, R.; Kondo, T.; Tatenuma, T.; Kawahara, T.; Komeya, M.; Ito, Y.; Hasumi, H.; et al. Impact of Chronic Kidney Disease Stages on Surgical and Functional Outcomes in Robot-Assisted Partial Nephrectomy for Localized Renal Tumors. *J. Robot. Surg.* **2024**, *18*, 109. https://doi.org/10.1007/s11701-024-01873-2.
28. Xiong, L.; Zou, X.; Luo, X.; Yin, S.; Huang, Y.; Ning, K.; Wen, D.; Zhou, Z.; Wang, J.; Li, Z.; et al. Longitudinal Changes in Renal Parenchymal Volume and Function Status After Partial Nephrectomy: A Retrospective Cohort Study. *Int. J. Surg.* **2024**, *110*, 984–991. https://doi.org/10.1097/JS9.0000000000000938.
29. Boylu, U.; Basatac, C.; Yildirim, U.; Onol, F.F.; Gumus, E. Comparison of Surgical, Functional, and Oncological Outcomes of Open and Robot-Assisted Partial Nephrectomy. *J. Minim. Access. Surg.* **2015**, *11*, 72–77. https://doi.org/10.4103/0972-9941.147699.
30. Chae, D.; Kim, N.Y.; Kim, K.J.; Park, K.; Oh, C.; Kim, S.Y. Predictive Models for Chronic Kidney Disease After Radical or Partial Nephrectomy in Renal Cell Cancer Using Early Postoperative Serum Creatinine Levels. *J. Transl. Med.* **2021**, *19*, 307. https://doi.org/10.1186/s12967-021-02976-2.
31. Kawase, K.; Enomoto, T.; Kawase, M.; Takai, M.; Kato, D.; Fujimoto, S.; Iinuma, K.; Nakane, K.; Kato, S.; Hagiwara, N.; et al. The Impact of Postoperative Renal Function Recovery after Laparoscopic and Robot-Assisted Partial Nephrectomy in Patients with Renal Cell Carcinoma. *Medicina* **2022**, *58*, 485. https://doi.org/10.3390/medicina58040485.

With this correction, the order of some references has been adjusted accordingly. The authors state that the scientific conclusions are unaffected. This correction was approved by the Academic Editor. The original publication has also been updated.
